# Detection of *TP53* R249 Mutation in Iranian Patients with Pancreatic Cancer

**DOI:** 10.1155/2013/738915

**Published:** 2013-12-30

**Authors:** Ashraf Mohamadkhani, Elnaz Naderi, Maryam Sharafkhah, Hamid Reza Fazli, Malihe Moradzadeh, Akram Pourshams

**Affiliations:** ^1^Liver and Pancreatobiliary Diseases Research Center, Digestive Diseases Research Institute, Tehran University of Medical Sciences, Tehran, Iran; ^2^Young Researchers Club, Ahar Branch, Islamic Azad University, Ahar, Iran; ^3^Faculty of Medicine, Mashhad University of Medical Science, Mashhad, Iran

## Abstract

The *TP53* gene encodes tumor protein p53 which play a major role in the etiology of pancreatic cancer. The important role of codon 249 of *TP53* for binding of p53 to its sequence-specific consensus site in DNA has been revealed by crystallography's studies, and mutation at this codon was detected in the plasma of some human cancers. The TP53 Mut assessor software within the International Agency for Research on Cancer (IARC) TP53 Database was performed to evaluate every possible mutation at codon 249. DNA was extracted from the plasma of 133 pancreatic cancer patients and 85 noncancer-bearing individuals. Exon 7 in *TP53* was amplified, and mutation at R249 was identified by the endonuclease cleavage of HaeIII. The group of patients showed a frequency of 11% (22 of 133 samples) R249 mutation compared to 3.5% (3 of 85 samples) in the group of control which was significant (*P* = 0.03). This mutation demonstrated statistically significant association with pancreatic cancer risk in unadjusted odds ratio (OR: 3.74, 95% CI: 1.1–13.2; *P* = 0.041); however when adjusted for confounding factors, it was marginally significant because of lower control samples. These findings demonstrate that mutation at R249 of *TP53* can be considered for increasing risk of pancreatic cancer that needs more research.

## 1. Introduction

Pancreatic ductal adenocarcinoma (PDAC), the most common cancer of the pancreas, is the fifth commonest cause of cancer-related mortality among industrialized countries [1]. There is not much data regarding incidence and mortality of pancreatic cancer in Middle East. However the cancer of pancreas is not in the top 10 of cancer-related deaths in Iran [2]. The aggressive nature of the PDAC is related to poor prognosis; therefore, most of the patients at the time of diagnosis are harboring unsuitable cancer that is extremely resistant to chemotherapy [3]. The incidence rate of pancreatic cancer has been stabilizing over the past two decades in many developed countries; however, this continues to increase in countries where the rate of pancreatic cancer was relatively low four decades ago, such as Japan [4]. In Iran, pancreatic cancer is diagnosed in advanced stage with no identified risk factors. Pancreatic cancer is primarily an environmental disease and attributed mostly to cigarette smoking [5]; however the pathogenesis of this cancer also involves various genetic alterations particularly genes that controlled the cell cycle [6, 7]. The *TP53 *(tumor protein p53) as a main tumor suppressor gene is mutated in most of the tumors [8, 9]. The p53 protein inhibits cancer formation through regulating several pathways involved in cellular functions for cell cycle arrest and apoptosis [10].

Mutations of the genome of *TP53* were currently at 60% of sporadic and 33% of familial pancreatic adenocarcinomas [11]. Mutations in the “hotspot” codons 175, 245, 248, 249, 273, and 282 cause distortions that create internal cavities or surface crevices in the protein scaffold leading to conformational changes in DNA binding surface [12]. Oligonucleotide array experiments showed that the *TP53* link to the promoter regions and regulate approximately of 300 different genes through its DNA binding domain [13]. Moreover, the important role of codon 249 of *TP53* for the function of this protein to bind to its sequence-specific consensus site in DNA has been studied by X-ray crystallography [14, 15]. Human cancer can be induced by environmental factors. For example, a substitution of serine for arginine in codon 249 of *TP53*, which is overcoming in areas with high exposure to aflatoxin B1, is associated with the incidence of hepatocellular carcinoma [16, 17].

Evaluation of mutation of *TP53 *might be used as a marker for incidence of cancers and may provide some clues about the significance of interaction of environmental and genetic factors. Several reports have offered that in some human cancers originated DNA from the tumor effectively can be extracted from the plasma or serum to study the specific missense mutation [14, 18]. Therefore, the aim of this study was to ascertain the prevalence of *TP53 *mutation at codon 249 in plasma in a case control study in patients with pancreatic ductal adenocarcinoma.

## 2. Patients and Methods

### 2.1. Patients

Blood samples were obtained from 133 pancreatic cancer patients and 85 noncancer-bearing individuals, and their plasma samples were stored at −70°C until use. Both case and control subjects were recruited between the years of 2010 and 2012. Cases were newly diagnosed patients attending at gastroenterology department of Shariati Hospital. The diagnosis of pancreatic cancer in patients was reconfirmed both histologically and clinically. Informed consent was obtained from all donors, and the protocol was approved by the institutional review board of the Shariati Hospital, Tehran University of Medical Science.

### 2.2. Evaluation of Mutations at Codon 249 of *p53* Gene

All mutations of *TP53* which was involved in human cancers are compiled in the International Agency for Research on Cancer (IARC) TP53 Database. The TP53 Mut Assessor software within IARC TP53 Database was used to evaluate the mutations at codon 249. This software as a novel stand-alone software allows the user to retrieve multiple information on every possible *TP53* mutant whether or not they have been described in human cancer. According to the acquired results by this software, every mutation in codon 249 (R249S/G/I/K/M/N/T/W) is able to inactive the function of p53 protein that shows the importance of codon 249 in folding, structure and function of p53 protein.

### 2.3. Amplification of Exon 7 of *TP53* and Mutation Detection

DNA was extracted from 200 *μ*L of plasma using a QiAamp blood kit (Qiagen Company) according to the manufacturer procedure. The standard PCR was performed in a total volume of 50 *μ*L as described previously [14] in which 50 ng template DNA, 10 pmol of each primer (sense: 5-CTTGCCACAGGTCTCCCCAA-3 and antisense: 5-AGGGGTCAGCGGCAAGCAGA-3), 0.25 mM of each dNTP, 2.5 mM MgCl_2_, 5 units of HotStarTaq polymerase (Qiagen), and the appropriate volume of H_2_O were used for amplification of a 236 bp region of exon 7 of the p53 gene in a “Touchdown” PCR program. The thermocycling conditions were 1 cycle of denaturation (5 min at 94°C), 20 cycles of denaturation (94°C for 45 s), annealing (63°C for 45 s, with –0.5°C per cycle), and extension (72°C for 1 min), followed by 30 cycles of denaturation (94°C for 45 s), annealing (60°C, 45 s), and extension (72°C for 1 min), and a final extension cycle of 72°C for 10 min.

The PCR products (*exon* 7) were digested with 10 units of the restriction endonuclease HaeIII (*Fermentas*, Hanover, MD), which cut within a GG*|*CC sequence encompassing codon 249 (AGG) in the wild-type sequence. However mutation at codon 249 would lose the HaeIII restriction endonuclease site. Digestion of DNA was performed in a total volume of 20 *μ*L in which 5 *μ*L PCR products were added to1 *μ*L HaeIII, 2 *μ*L 10 x buffer, and 12 *μ*L ddH_2_O for 2-hour incubation at 37°C. The genotype screening was performed simultaneously for cases and controls. The detected mutations were validated by DNA sequencing analysis.

### 2.4. Statistical Analysis

All statistical analyses were performed with the Statistical Program for Social Sciences (SPSS, version 19 for Windows; SPSS Inc., Chicago, Il). The mean ± standard deviation (SD) was considered for continuous variables, and the frequency (%) was reported for categorical variables. Demographic variables were compared between two groups with independent *t*-test or pearson's chi-square test as appropriate. Simple and multivariable logistic regression was used to assess the effect of independent variables on *TP53 *mutation at codon 249 as a dependent variable. A *P* value of ≤0.05 denoted significance.

## 3. Results

### 3.1. PCR-RFLP Analysis of *TP53* at Codon 249

To examine the *TP53* mutation of codon 249 in patients with pancreatic cancer and healthy controls, the final PCR fragments of p53 exon 7 with 236 bp in size were digested by HaeIII restriction enzyme. The recognition site GGCC for HaeIII restriction enzyme in wild-type sequences of exon 7 represents the arginine-encoding allele in codon 249 and generates a separated 91 bp fragment beside the fragment of 66 bp and also fragments of 37 bp, 30 bp, and 12 bp. However in individuals with the asb-arginine (each amino acid except for arginine) a distinct fragment of 157 bp plus the fragments of 37 bp, 30 bp, and 12 bp in size has been recognized. Two patients showed the homozygous pattern for the mutation (both alleles were mutated) while the other 14 patients were heterozygous for the same mutation. All the three mutations found in the control populations had heterozygous pattern.

### 3.2. Mutation Distribution among Patients and Control

The characteristics of all the subjects in two groups of case and control were presented in Table 1. There were a total of 133 patients with mean age of 64 ± 9 years old compared to eighty-five noncancerous individuals with mean age of 64 ± 11 years old (*P* = 0.7) that did not differ between the patients and the controls. However there was significant difference in gender between the patients and the controls (*P* = 0.016) which was because of lower control sample size. When we matched eighty-five individuals of both cases and control, there was no significant difference in sex between groups. Proportion of smokers also did not show significant difference between two groups. The relative frequency of *TP53* mutation at codon 249 was 11.1% (16 of 133 samples) in patients with pancreatic cancer compared to 3.5% (3 of 85 samples) in the group of control which was significant (*P* = 0.03). There was also no difference in familial history of cancer between two groups (Table 1).

The odds ratios for the mutation of *TP53* at codon 249 are shown in Table 2. This mutation demonstrated statistically significant association with pancreatic cancer risk (OR 3.74: 95% CI 1.1–13.2; *P* = 0.041). Remarkably despite the significant difference in the distribution of gender between the patients and the controls, when R249 mutant was adjusted for other characteristics, there was no significant association with pancreatic cancer risk (adjusted OR: 3.12, 95% CI: 0.84–11.6; *P* = 0.089). The wide variation of confidence interval and *P* value could be refined when the sample size of cases and controls was revised.

## 4. Discussion

In pancreatic cancer, the progression from minimally dysplastic epithelium to invasive carcinoma accompanied with the consecutive accumulation of mutations that include activation of oncogenes and inactivation of the tumor-suppressor genes like *TP53* [10, 11, 19]. The results of this study identified the higher frequency of *TP53* mutation at codon 249 in pancreatic cancer patients. We found that the R249 mutation increased the risk of cancer with no significant difference in the age and gender at cancer diagnosis.

The results from recent study by Biankin et al., which performed exome sequencing and copy number analysis, defined the significant mutations in 16 genes. Their results not only are reaffirming known mutations in individual pancreatic cancers but also by using GeneGO15 showed the importance of mutations in *TP53* and the mechanisms that are important in G1/S checkpoint machinery and apoptosis [20]. Moreover, earlier study on molecular pathology of human pancreatic cancer by Ruggeri et al. with regard to the role of known tumor-suppressor genes revealed missense mutations in codons 181, 220, 248, 249, 265, 272, and 273 and suggested the important features of these mutations in the development of human pancreatic cancer [21]. Mutant *TP53* has extra functions above loss of normal activity of p53; this stems from the study by Morton et al. that evidenced the function of *TP53* mutations in pancreatic cancer to escape from growth arrest/senescence and a promotion of metastasis [22].

The residue of R249 is one of the most frequent mutation in human cancers which promotes the transition of G0 to G1 and/or M to G1 during the cell cycle and is supported by the mutant form of p53 protein which has a longer half-life [23]. Nonetheless the mutation in this residue is not in direct contact with DNA; however it has an important role in stabilizing the DNA-binding structure of p53 and therefore can be attributed as structural mutant [16, 24]. These types of mutants change the conformation of the protein that affects the overall architecture of the DNA-binding surface and its function [24].

The results obtained by Rui et al. revealed that the carboxyl half of the DNA-binding domain (DBD) of p53, including the residue of R249, is responsible for direct interaction with Axin [25]. This protein and p53 are tumor suppressors that control cell growth, apoptosis, and development [25, 26]. Beside the interaction with p53 via HIPK2 protein, Axin can also directly interact with p53 protein through a separate domain to stimulate p53-dependent reporter transcription [25]. It is possible that the mutation in R249 residue disturb the function of Axin tumor suppressor for facilitating the p53 function. Another possible explanation for the R249 mutation is the dietary exposure to the fungal toxin, the aflatoxin B1 (AFB1). The R249 mutation is more frequent in patients with hepatocellular carcinoma in a region with high levels of AFB exposure in their diet. From general aspect, mutation at codon 249 could increase *TP53* mRNA expression and result in overexpression of mutant p53 proteins [27].

Of the sixteen R249 mutations that were found in our patients group, 14 showed positivity in the heterozygous form that exhibit deficiency of one allele which is sufficient for a lethal outcome. It has been proposed that the development of cancers in the exposure to carcinogens is even more accurate and faster when combined with heterozygosity for p53 (deficiency of one allele) [28]. However, because of the source of plasma DNA from different cells, the heterozygous cases could have the homozygous/heterozygous mixed pattern.

## 5. Conclusion

This study showed a higher frequency of R249 mutation in patients with pancreatic cancer. Also the presence of the R249 *TP53 *mutation in the plasma of patients with pancreatic cancer and also in healthy subjects may reflect chronic exposure to high levels of AFB1. Further studies are needed to better define the role of *TP53* alterations in pancreatic cancers and possibly to understand the impact of mutations on cancer prognosis and outcomes.

## Figures and Tables

**Table 1:** Characteristics of the study population.

	Case (*n* = 133)	Control (*n* = 85)	*P* value
Age (mean ± SD)	64.2 ± 9.6	64.7 ± 11.6	0.739
Gender (female/male)	53/80	48/37	0.016
Smoking (yes/no)	25/108	23/62	0.151
Familial history of cancer (yes/no)	28/105	26/59	0.112
TP53 mutation at Codon 249 (R249) (*n* (%))	16 (11%)	3 (3.5%)	0.030

**Table 2:** Unadjusted and adjusted odds ratios (OR) and 95% confidence intervals (95% CI) for the TP53 mutation at codon 249 status in cases and control subjects.

	Univariable model		Multivariable model	
	Unadjusted OR (95% CI)	*P* value	Adjusted OR (95% CI)*	*P* value
R249 wild type (%)	1		1	
R249 mutant (%)	3.74 (1.1–13.2)	0.041	3.12 (0.84–11.6)	0.089

*Adjusted odds ratio for gender, age, smoking, and familial history of cancer.
